# Asymptomatic Pellet Migration to the Heart: Report of a Case and Update on Proper Management

**DOI:** 10.1155/2014/257614

**Published:** 2014-11-06

**Authors:** George Galyfos, Konstantinos Palogos, Nikolaos Kavouras

**Affiliations:** Department of General Surgery, General Hospital of Chalkis, 48 Gazepi Street, 34100 Euboea, Greece

## Abstract

We report a case of a 42-year-old male patient who was transferred to our emergency department suffering from a gunshot wound in his left lateral thigh. The patient was haemodynamically stable, and the physical examination of the abdomen and thorax was unremarkable. There was no obvious exit point and there were no other injuries. The radiologic control of the left thigh showed an intact femur and multiple pellets within the adjacent soft tissues. Routine X-ray evaluation of the thorax revealed a small-sized round object of metal density—possibly a migrated pellet—in the proximity of the right heart atrium. Computed tomography imaging confirmed this finding and showed no other cardiac or mediastinal injury. Ultrasonography of the heart was unremarkable as well. The patient was managed conservatively for the discovered pellet, and remained asymptomatic throughout the entire hospital stay, and 6 months after the discharge. Pellet migration or embolism should be suspected in any gunshot victim without a corresponding exit wound or when the signs and symptoms do not correlate with the suspected course of the missile. Conservative management remains the first choice in asymptomatic patients, although close monitoring at first and regular observation after discharge are indicated.

## 1. Introduction

Penetrating gunshot wounds of the thorax remain a major surgical challenge and show a high incidence, especially in warzones and societies with increasing crime rates [1]. However, the discovery of pellets or bullets within the thoracic cavity and especially the heart, after migration from distant entry sites of the body, is unusual [2]. We report a case of a 42-year-old male patient with a gunshot wound in the left lower extremity and a pellet migration in the right atrium of the heart.

## 2. Case Presentation

A 42-year-old male was transferred to our emergency department having been shot with a hunter's rifle in his left lower thigh. The patient was haemodynamically stable (Blood Pressure: 140/80 mm Hg; Heart Rate: 95/min; SO_2_: 100%; Glasgow scale: 15/15). The physical examination revealed an open wound (almost 10 cm × 6 cm in size) at the lateral side of his left thigh, with multiple pellets visible inside and around the wound. There were no other visible injuries. The auscultation of the thorax did not reveal any abnormal respiratory or cardiac sounds. The abdominal examination revealed no sensitivity or abnormal bowel sounds. There was significant tissue damage (mainly subcutaneous and muscular tissues) at the entry point, without any visible exit point and without injury of major vascular structures. There were no signs of ischemia or neurological deficits at the time. The medical history of the patient was unremarkable as well.

The laboratory findings revealed leucocytosis (WBC: 14,700; NEU: 81%), anaemia (HCT: 33%; MCV: 83.9 fL), and a normal platelet number (PLT: 222,000). The remaining laboratory studies were as follows: glucose: 120 mg/dL; urea: 19.6 mg/dL; creatinine: 0.73 mg/dL; CPK: 10,900 U/L; K^+^: 3.83 mmol/l; Na^+^: 132 mmol/l. Clotting times were normal as well. The patient received intravenous fluids, prophylaxis against tetanus, wide spectrum antibiotics (cefuroxime and clindamycin i.v.), antithrombotic prophylaxis, and adequate analgesia.

**Figure 1:**
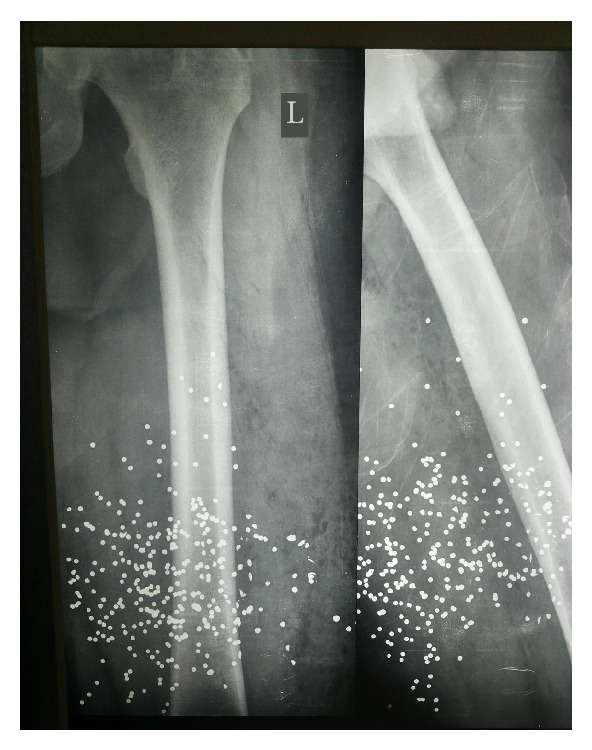
X-ray of the left femoral bone. Multiple pellets are visible in the surrounding soft tissues. No bone fracture.

**Figure 2:**
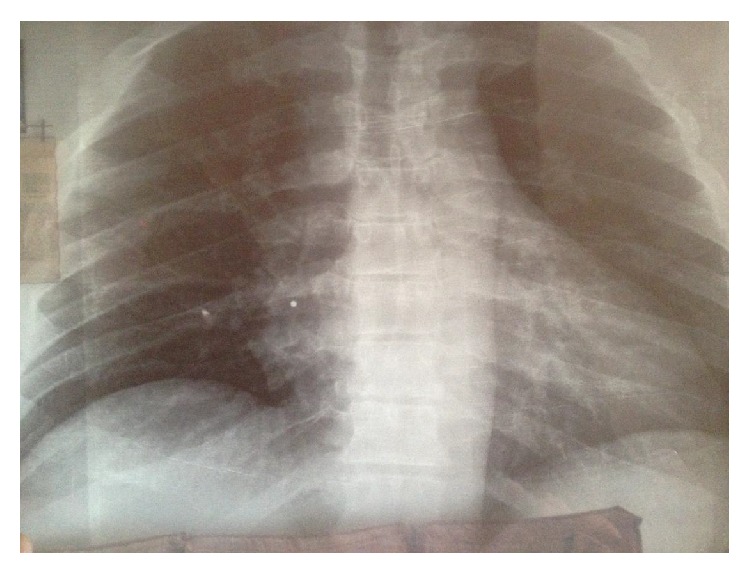
X-ray of the thorax. A small-sized round object of metal density (pellet) lies in the proximity of the right atrium.

**Figure 3:**
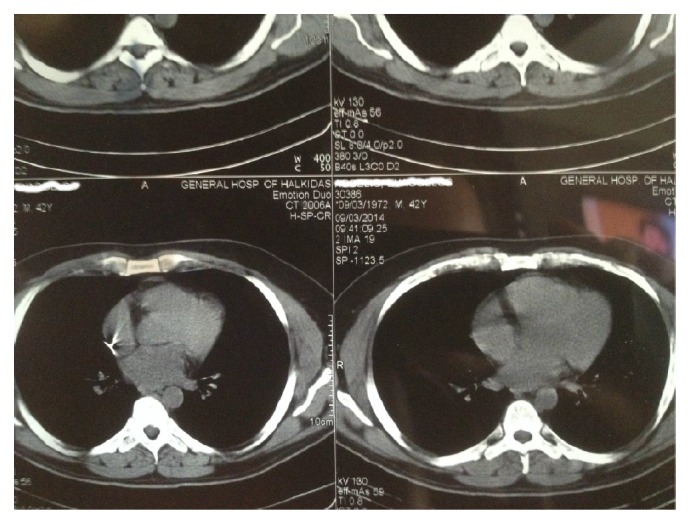
Computed tomography (CT) of the thorax. A small-sized metallic artifact lies at the opening of the right atrium of the heart. No other cardiac or mediastinal injury.

The radiologic control of the left thigh showed an intact femur and multiple pellets inside the adjacent soft tissues (Figure 1). The X-ray images of the thorax did not reveal any injury, although a small and round object of metal density was detected in the proximity of the right atrium (Figure 2). The ultrasonographic control of the heart was unremarkable. However, a computed tomography (CT) study of the thorax was ordered that illustrated a single pellet at the entry of the right atrium, without any other cardiac or mediastinal injury (Figure 3). The most plausible explanation for the intracardiac pellet was intravascular migration from the femoral veins to the heart via the inferior vena cava.

The patient underwent surgical debridement of the wound, where multiple pellets were removed and adjacent soft tissues were explored. Conservative management with antibiotics and serial scanning to monitor further bullet migration was favoured over surgical extraction of the intracardiac pellet. This decision was based on the patient being asymptomatic, the pellet being on the right side of the heart, and clinical experience of previous similar cases. After consulting with a cardiac surgeon as well, the patient was admitted into our surgical department for further observation and monitoring.

The patient remained asymptomatic throughout the admission and was discharged after 5 days. Out-patient reevaluation with X-rays at 6 weeks and 6 months after discharge showed that the pellet remained at the same location. The patient remains stable after 6 months and without history of embolic events.

## 3. Discussion

Since the first documented case by Davis in 1834, many cases of foreign body embolization have been described, with bullets accounting only for 0.3% of the responsible artifacts [3]. Migration of a bullet to a distant part of the body after a gunshot is rarely observed in the clinical setting, and migration to the heart is even rarer [3].

There are usually no clear symptoms or signs from migration of a bullet. Venous emboli are often an occult phenomenon and may remain unrecognised until migration leads to vascular injury or flow obstruction [4]. Therefore, the bullet can be easily missed and sometimes identified during a review examination. Hence, the pellet in our patient was discovered during a routine X-ray of the thorax. Bullet emboli have been incidentally identified months to decades after a reported gunshot [5]. Furthermore, the site of origin can be located anywhere in the body, and the bullet may migrate via several routes. It may migrate locally along the intermuscular space or lumen, or it may migrate along the vein or artery to a distant site [6]. Migration of a bullet from the abdomen to the right ventricle after a gunshot causing bowel perforation has been reported by Palmen et al. [7]. Hussein et al. reported the identification of a migrated pellet within the right ventricle of a patient who sustained a gunshot wound in the leg 10 years prior to presenting with shortness of breath and localised chest pain [8]. Our patient's entry point was located in the lower extremity as well.

Traumatic foreign objects invade the vasculature via direct propulsion into the lumen or erosion into the vessel wall [3]. Subsequent embolization exists in two types: 80% being arterial and 20% being venous [9]. Venous embolization from the peripheral vasculature to the vena cava, right ventricle, or pulmonary arteries can cause symptoms such as perforation, further embolization, endocarditis, septic emboli, dyspnoea, haemoptysis, and chest pain in approximately 30% of patients [9, 10]. However, our patient was fully asymptomatic. Currently, two rare subtypes of venous embolization exist [9]. The first is retrograde embolization where the object moves against the normal direction of blood flow and this occurs in 15% of venous cases. The second is paradoxical embolization, where the object moves from the venous circulation, through a right-to-left shunt, traverses into the arterial circulation, and behaves similarly to an arterial embolus.

Regarding management, surgical intervention for the treatment of symptomatic patients has been clear so far. Reasons for removal of intracardiac pellets include avoidance of major venous obstruction, endocarditis, arrhythmias, myocardial irritability, valvular dysfunction, and delayed migration [9]. Objects >5 mm in diameter, or an irregular shape, are also indications for removal [10]. However, the foreign object in our case was a small-sized round pellet. Shannon et al. [11] reviewed 102 cases of gunshot injuries and found that a bullet retained in a blood vessel is associated with complication incidence of 25% and death rate of 6%. However, the incidence of complications is reduced to 1%-2% if the bullet is removed. Therefore, the authors recommended surgical removal of the bullet. Moreover, the evolution of endovascular techniques has introduced a new, less invasive therapeutic method [12].

Asymptomatic lung emboli, however, do not usually lead to serious sequelae [9]. Therefore, most centres favour conservative management unless the patient acutely deteriorates. Marchaland et al. [13] underline that many problems must be highlighted: mechanics of entry into the heart (own velocity, venous flow), topographic diagnosis (chest X-ray, transthoracic or transoesophageal ultrasound, and CT-scan), local outcomes of this projectile (local erosion, clot, and endocarditis), destination of a new migration (pulmonary embolism, left heart), indications of extraction, and supervision. Extraction should be systematic only in the case of a patent foramen ovale where clinical outcomes are most serious or in the event of complications.

Our patient was successfully treated with observation and without any intervention. Other authors support the conservative management as well [14]. Kortbeek et al. present a total of 32 cases reported since 1966, with no deaths [15]. Fourteen of these patients were managed only with observation and five patients were followed up with no resulting complications noted. The authors conclude that conservative management of selected cases with pulmonary artery bullet emboli may be warranted in light of the risks of extraction. Even in children, management decisions regarding thoracic/cardiac pellet gun injuries must be based on the presentation and stability of the patient and the location of the retained pellet [14].

## 4. Conclusions

Pellet migration or embolism should be suspected in any patient who has a gunshot wound without a corresponding exit wound, when the signs and symptoms do not correlate with those expected from the suspected course of the missile or when radiologic studies show that missile location is deviating from the path of penetration. Conservative management remains the first choice in asymptomatic patients with migrating pellets to the heart, although close monitoring at first and regular observation after discharge are indicated.
